# Supplementary material for: Machine Learning Accelerates Crystallization for Structure Determination
Source: Angew Chem Int Ed Engl. 2026 May 4;65(25):e1218503. doi: 10.1002/anie.1218503 (PMC13266958; doi:10.1002/anie.1218503)

checkCIF/PLATON report

Structure factors have been supplied for datablock(s) lcz102

THIS REPORT IS FOR GUIDANCE ONLY. IF USED AS PART OF A REVIEW PROCEDURE FOR PUBLICATION, IT SHOULD NOT REPLACE THE EXPERTISE OF AN EXPERIENCED CRYSTALLOGRAPHIC REFEREE.

No syntax errors found. CIF dictionary Interpreting this report

Datablock: lcz102

Bond precision:	C-C = 0.0212 A		Wavelength=1.54184
Cell:	a=13.4294(4)	b=18.0985(4)	c=15.4233(4)
	alpha=90	beta=110.490(3)	gamma=90
Temperature:	100 K		

	Calculated	Reported
Volume	3511.50(17)	3511.50(17)
Space group	P 21/c	P 1 21/c 1
Hall group	-P 2ybc	-P 2ybc
Moiety formula	C27 H11 Ag3 Br F18 I N6	C15 H3 Ag3 F18 N6, C12 H8 Br I
Sum formula	C27 H11 Ag3 Br F18 I N6	C27 H11 Ag3 Br F18 I N6
Mr	1291.83	1291.84
Dx,g cm-3	2.444	2.444
Z	4	4
Mu (mm-1)	22.729	22.730
F000	2424.0	2424.0
F000'	2430.83	
h,k,lmax	17,23,19	16,22,19
Nref	7547	7257
Tmin,Tmax	0.066,0.033	0.327,1.000
Tmin'	0.011	

Correction method= # Reported T Limits: Tmin=0.327 Tmax=1.000
AbsCorr = MULTI-SCAN

Data completeness= 0.962 Theta(max)= 78.623

R(reflections)= 0.0877(6068) wR2(reflections)= 0.2445(7257)

S = 1.051 Npar= 505

The following ALERTS were generated. Each ALERT has the format
test-name_ALERT_alert-type_alert-level.
Click on the hyperlinks for more details of the test.

Alert level A

PLAT971_ALERT_2_A Check Calcd Resid. Dens. 0.20Ang From Br05 5.06 eA-3

Author Response: This Alert is due to presence of residual density in the presence of heavy metal atom.

PLAT971_ALERT_2_A Check Calcd Resid. Dens. 1.32Ang From F01F 3.81 eA-3

Author Response: This Alert is due to presence of residual density in the presence of heavy metal atom.

Alert level B

PLAT342_ALERT_3_B Low Bond Precision on C-C Bonds .... 0.02116 Ang.

Author Response: Disordered structure.

PLAT971_ALERT_2_B Check Calcd Resid. Dens. 0.99Ang From N00J 3.30 eA-3

Author Response: This Alert is due to presence of residual density in the presence of heavy metal atom.

PLAT971_ALERT_2_B Check Calcd Resid. Dens. 1.12Ang From F01G 3.05 eA-3

Author Response: This Alert is due to presence of residual density in the presence of heavy metal atom.

PLAT971_ALERT_2_B Check Calcd Resid. Dens. 1.04Ang From N00B 2.75 eA-3

Author Response: This Alert is due to presence of residual density in the presence of heavy metal atom.

```
PLAT971_ALERT_2_B Check Calcd Resid. Dens.  1.01Ang From F01F           2.66 eA-3
```

Author Response: This Alert is due to presence of residual density in the presence of heavy metal atom.

```
PLAT972_ALERT_2_B Check Calcd Resid. Dens.  0.61Ang From I04           -2.82 eA-3
```

Author Response: This Alert is due to presence of residual density in the presence of heavy metal atom.

```
PLAT972_ALERT_2_B Check Calcd Resid. Dens.  0.66Ang From I04           -2.57 eA-3
```

Author Response: This Alert is due to presence of residual density in the presence of heavy metal atom.

```
PLAT972_ALERT_2_B Check Calcd Resid. Dens.  0.55Ang From I04           -2.52 eA-3
```

Author Response: This Alert is due to presence of residual density in the presence of heavy metal atom.

Alert level C

```
DIFMX02_ALERT_1_C  The maximum difference density is > 0.1*ZMAX*0.75
            The relevant atom site should be identified.
PLAT042_ALERT_1_C Calc. and Reported MoietyFormula Strings  Differ     Please Check
              Calc: C27 H11 Ag3 Br F18 I N6
              Rep.: C15 H3 Ag3 F18 N6, C12 H8 Br I
PLAT097_ALERT_2_C Large Reported Max.  (Positive) Residual Density       4.80 eA-3
PLAT213_ALERT_2_C Atom C00T            has ADP max/min Ratio .....        3.6 prolat
PLAT213_ALERT_2_C Atom C010            has ADP max/min Ratio .....        3.4 prolat
PLAT234_ALERT_4_C Large Hirshfeld Difference F012     --C01K     .       0.21 Ang.
PLAT234_ALERT_4_C Large Hirshfeld Difference F01F     --C01J     .       0.19 Ang.
PLAT790_ALERT_4_C Centre of Gravity not Within Unit-Cell: Resd.  #          1 Note
              C27 H11 Ag3 Br F18 I N6
PLAT906_ALERT_3_C Large K Value in the Analysis of Variance ......      6.834 Check
PLAT906_ALERT_3_C Large K Value in the Analysis of Variance ......      2.368 Check
PLAT911_ALERT_3_C Missing FCF Refl Between Thmin & STh/L=    0.600         14 Report
                9   2  3,    3  6  7,  -3  8  7,   5 11 10, -13  9 12, -10  9 15,
               -9   9 15,  -4  0 16, -10  8 16,  -9  8 16,  -5 10 16,  -9  4 17,
               -4   7 17,  -5  8 17,
PLAT971_ALERT_2_C Check Calcd Resid. Dens.  0.92Ang From N00C           2.28 eA-3
```

Author Response: This Alert is due to presence of residual density in the presence of heavy metal atom.

```
PLAT971_ALERT_2_C Check Calcd Resid. Dens.  1.14Ang From I04            1.70 eA-3
```

Author Response: This Alert is due to presence of residual density in the presence of heavy metal atom.

```
PLAT971_ALERT_2_C Check Calcd Resid. Dens.  1.22Ang From I04            1.68 eA-3
```

Author Response: This Alert is due to presence of residual density in the presence of heavy metal atom.

```
PLAT971_ALERT_2_C Check Calcd Resid. Dens.  1.14Ang From Ag00           1.65 eA-3
```

Author Response: This Alert is due to presence of residual density in the presence of heavy metal atom.

```
PLAT971_ALERT_2_C Check Calcd Resid. Dens.  1.23Ang From Ag03           1.61 eA-3
```

Author Response: This Alert is due to presence of residual density in the presence of heavy metal atom.

```
PLAT971_ALERT_2_C Check Calcd Resid. Dens.  1.08Ang From F01G           1.58 eA-3
```

Author Response: This Alert is due to presence of residual density in the presence of heavy metal atom.

```
PLAT971_ALERT_2_C Check Calcd Resid. Dens.  1.30Ang From I04            1.56 eA-3
```

Author Response: This Alert is due to presence of residual density in the presence of heavy metal atom.

```
PLAT977_ALERT_2_C Check Negative Difference Density on H00X      .      -0.40 eA-3
PLAT977_ALERT_2_C Check Negative Difference Density on H00Z      .      -0.34 eA-3
```

Alert level G

```
PLAT003_ALERT_2_G Number of Uiso or U(i,j)  Restrained non-H-Atoms         8 Report
PLAT072_ALERT_2_G SHELXL First  Parameter in WGHT  Unusually Large      0.11 Report
PLAT083_ALERT_2_G SHELXL Second Parameter in WGHT  Unusually Large    152.84 Why ?
PLAT177_ALERT_4_G The CIF-Embedded .res File Contains DELU Records         1 Report
PLAT178_ALERT_4_G The CIF-Embedded .res File Contains SIMU Records         1 Report
PLAT186_ALERT_4_G The CIF-Embedded .res File Contains ISOR Records         3 Report
PLAT232_ALERT_2_G Hirshfeld Test Diff (M-X)  I04      --Ag00     .       6.9 s.u.
PLAT232_ALERT_2_G Hirshfeld Test Diff (M-X)  I04      --Ag02     .      11.6 s.u.
PLAT242_ALERT_2_G Low    'MainMol' Ueq as Compared to Neighbors of      C018 Check
PLAT242_ALERT_2_G Low    'MainMol' Ueq as Compared to Neighbors of      C01A Check
PLAT242_ALERT_2_G Low    'MainMol' Ueq as Compared to Neighbors of      C01B Check
PLAT242_ALERT_2_G Low    'MainMol' Ueq as Compared to Neighbors of      C01I Check
```

```
PLAT242_ALERT_2_G Low    'MainMol' Ueq as Compared to Neighbors of       C01J Check
PLAT242_ALERT_2_G Low    'MainMol' Ueq as Compared to Neighbors of       C01K Check
PLAT434_ALERT_2_G Short Inter HL..HL Contact I04      ..F008     .       3.34 Ang.
                                          3-x,2-y,2-z  =      3_877 Check
PLAT434_ALERT_2_G Short Inter HL..HL Contact F00E     ..F00M     .       2.82 Ang.
                                        x,5/2-y,-1/2+z  =      4_585 Check
PLAT434_ALERT_2_G Short Inter HL..HL Contact F00O     ..F01F     .       2.84 Ang.
                                       1+x,5/2-y,1/2+z  =      4_686 Check
PLAT720_ALERT_4_G Number of Unusual/Non-Standard Labels .........          67 Note
              Ag00    Ag02    Ag03    I04     Br05    F006    F007    F008
              F009    F00A    N00B    N00C    F00D    F00E    F00F    F00G
              F00H    N00I    N00J    N00K    N00L    F00M    F00N    F00O
              C00P    H00P    C00Q    H00Q    C00R    C00S    H00S    C00T
              C00U    H00U    C00V    C00W    C00X    H00X    C00Y    C00Z
              H00Z    C010    H010    C011    H011    F012    C013    H013
              C014    H014    C015    F016    C017    C018    C019    H019
              C01A    C01B    F01C    C01D    C01E    F01F    F01G    C01H
              C01I    C01J    C01K
PLAT860_ALERT_3_G Number of Least-Squares Restraints ............          51 Note
PLAT883_ALERT_1_G Absent Datum for _atom_sites_solution_primary ..     Please Do !
PLAT912_ALERT_4_G Missing # of FCF Reflections Above STh/L=  0.600        270 Note
PLAT933_ALERT_2_G Number of HKL-OMIT Records in Embedded .res File          3 Note
               -5  8 17,  -5 10 16,  -4  7 17,
PLAT941_ALERT_3_G Average HKL Measurement Multiplicity ..........         2.5 Low
PLAT969_ALERT_5_G The 'Henn et al.' R-Factor-gap value ..........       4.083 Note
              Predicted wR2: Based on SigI**2  5.99 or SHELX Weight 23.26
PLAT978_ALERT_2_G Number C-C Bonds with Positive Residual Density.          0 Info
```

```
   2 ALERT level A = Most likely a serious problem - resolve or explain
   8 ALERT level B = A potentially serious problem, consider carefully
  20 ALERT level C = Check. Ensure it is not caused by an omission or oversight
  25 ALERT level G = General information/check it is not something unexpected

   3 ALERT type 1 CIF construction/syntax error, inconsistent or missing data
  37 ALERT type 2 Indicator that the structure model may be wrong or deficient
   6 ALERT type 3 Indicator that the structure quality may be low
   8 ALERT type 4 Improvement, methodology, query or suggestion
   1 ALERT type 5 Informative message, check
```

It is advisable to attempt to resolve as many as possible of the alerts in all categories. Often the minor alerts point to easily fixed oversights, errors and omissions in your CIF or refinement strategy, so attention to these fine details can be worthwhile. It is up to the individual to critically assess their own results and, if necessary, seek expert advice.

Validation response form

Please find below a validation response form (VRF) that can be filled in and pasted into your CIF.

```
# start Validation Reply Form
_vrf_DIFMX02_lcz102
;
PROBLEM: The maximum difference density is > 0.1*ZMAX*0.75
RESPONSE: ...
```

```
;
_vrf_PLAT042_lcz102
;
PROBLEM: Calc. and Reported MoietyFormula Strings  Differ     Please Check
RESPONSE: ...
;
_vrf_PLAT097_lcz102
;
PROBLEM: Large Reported Max.  (Positive) Residual Density       4.80 eA-3
RESPONSE: ...
;
_vrf_PLAT213_lcz102
;
PROBLEM: Atom C00T            has ADP max/min Ratio .....        3.6 prolat
RESPONSE: ...
;
_vrf_PLAT234_lcz102
;
PROBLEM: Large Hirshfeld Difference F012     --C01K     .       0.21 Ang.
RESPONSE: ...
;
_vrf_PLAT790_lcz102
;
PROBLEM: Centre of Gravity not Within Unit-Cell: Resd.  #          1 Note
RESPONSE: ...
;
_vrf_PLAT906_lcz102
;
PROBLEM: Large K Value in the Analysis of Variance ......      6.834 Check
RESPONSE: ...
;
_vrf_PLAT911_lcz102
;
PROBLEM: Missing FCF Refl Between Thmin & STh/L=    0.600         14 Report
RESPONSE: ...
;
_vrf_PLAT977_lcz102
;
PROBLEM: Check Negative Difference Density on H00X      .      -0.40 eA-3
RESPONSE: ...
;
# end Validation Reply Form
```

PLATON version of 17/09/2025; check.def file version of 17/09/2025

---

duplicate check

No duplication found

Datablock lcz102 - ellipsoid plot

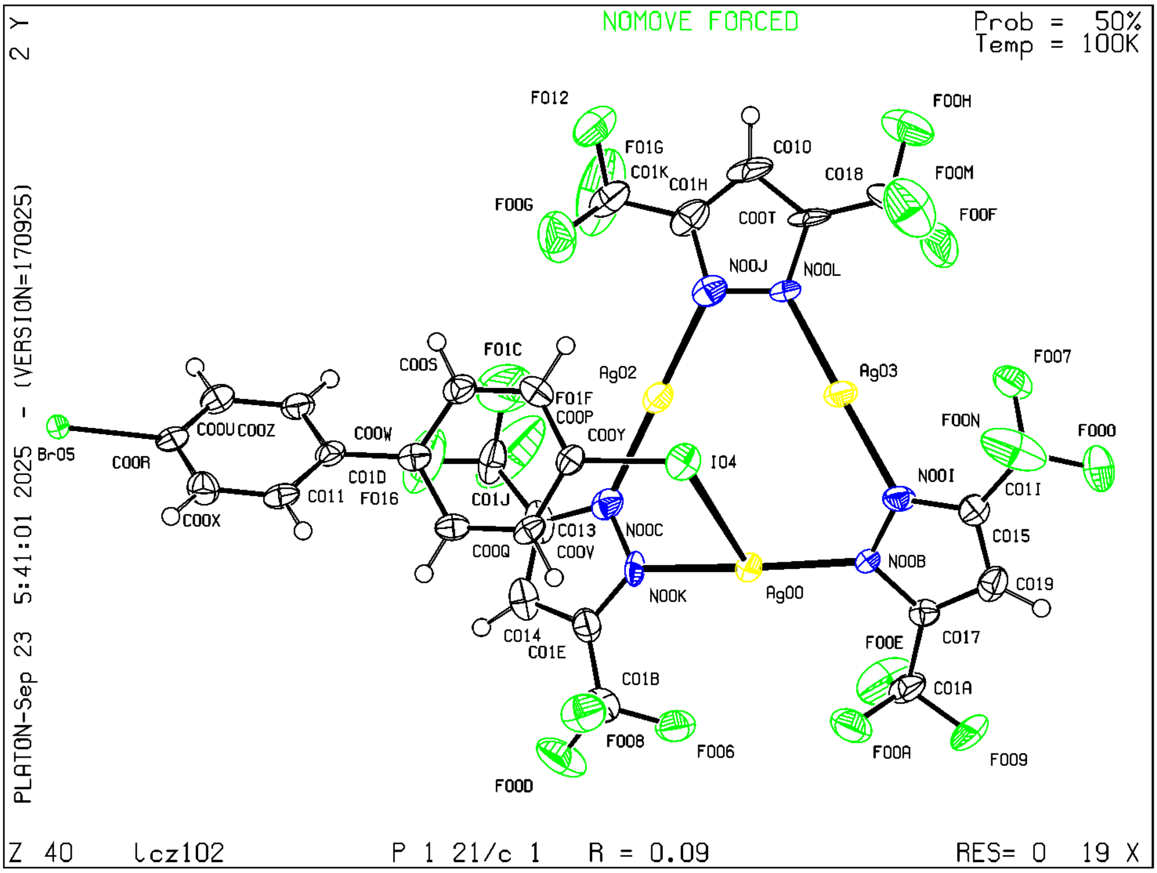

Supplement: Supplementary file 2 — Supporting File 2: anie72462‐sup‐0002‐DataFile.zip. [file ANIE-65-e1218503-s002.zip › supplementary_materials_crystallographic_data submit.zip!/cif_and_checkcif(1-114)/Ag3Pz3_102/Ag3Pz3_102_checkcif.pdf]
